# Comparison of Test Statistics of Nonnormal and Unbalanced Samples for Multivariate Analysis of Variance in terms of Type-I Error Rates

**DOI:** 10.1155/2019/2173638

**Published:** 2019-07-18

**Authors:** Can Ateş, Özlem Kaymaz, H. Emre Kale, Mustafa Agah Tekindal

**Affiliations:** ^1^Department of Biostatistics, Van Yüzüncü Yıl University, Van, Turkey; ^2^Department of Statistics, Ankara University, Ankara, Turkey; ^3^Department of Interdisciplinary Neuroscience, Ankara University, Ankara, Turkey; ^4^Department of Biostatistics, Selçuk University, Konya, Turkey

## Abstract

In this study, we investigate how Wilks' lambda, Pillai's trace, Hotelling's trace, and Roy's largest root test statistics can be affected when the normal and homogeneous variance assumptions of the MANOVA method are violated. In other words, in these cases, the robustness of the tests is examined. For this purpose, a simulation study is conducted in different scenarios. In different variable numbers and different sample sizes, considering the group variances are homogeneous (*σ*_12_ = *σ*_22_ = ⋯ = *σ*_*g*2_) and heterogeneous (increasing) (*σ*_12_ < *σ*_22_ < ⋯<*σ*_*g*2_), random numbers are generated from Gamma(4-4-4; 0.5), Gamma(4-9-36; 0.5), Student's *t*(2), and Normal(0; 1) distributions. Furthermore, the number of observations in the groups being balanced and unbalanced is also taken into account. After 10000 repetitions, type-I error values are calculated for each test for *α* = 0.05. In the Gamma distribution, Pillai's trace test statistic gives more robust results in the case of homogeneous and heterogeneous variances for 2 variables, and in the case of 3 variables, Roy's largest root test statistic gives more robust results in balanced samples and Pillai's trace test statistic in unbalanced samples. In Student's *t* distribution, Pillai's trace test statistic gives more robust results in the case of homogeneous variance and Wilks' lambda test statistic in the case of heterogeneous variance. In the normal distribution, in the case of homogeneous variance for 2 variables, Roy's largest root test statistic gives relatively more robust results and Wilks' lambda test statistic for 3 variables. Also in the case of heterogeneous variance for 2 and 3 variables, Roy's largest root test statistic gives robust results in the normal distribution. The test statistics used with MANOVA are affected by the violation of homogeneity of covariance matrices and normality assumptions particularly from unbalanced number of observations.

## 1. Introduction

Variance analysis is a method used to test whether there is a statistical difference between three or more group means. Multivariate analysis of variance (MANOVA) is the extended version of univariate analysis of variance (ANOVA). However, MANOVA is a statistical method that examines the effect of two or more independent variables on two or more dependent variables [1]. MANOVA is a method that can be used when several measurements are made on each person or object in one or more samples. Measurements are taken based upon the response variables. Hence, MANOVA data format, different from ANOVA, can be considered as a vector [2]. The test statistics for MANOVA give a measure of the overall likelihood of picking two or more random vectors of means [2, 3].

MANOVA has three main assumptions as in all parametric tests. The first one is the assumption that observations are independent of each other. This assumption explains that the sample is completely random. The second assumption is that the dependent variables have a multivariate normal distribution in group. The third assumption is the homogeneity of variances. In this test, since there is more than one dependent variable, not only the equality of the variances between the groups should be ensured but also the equality of the covariance between the dependent variables must be sustained. For this, the variance-covariance matrix is used.

In his study, Olson [4] has analyzed a total of 6 test statistics including Wilks' lambda, Pillai's trace, Hotelling's trace, and Roy's largest root test statistics where the number of variables is 2, 3, 6, and 10; the number of groups is 2, 3, 6, and 10; and sample size is 5, 10, and 50, for type-I and type-II errors in 1000 repetitions. In addition, in [5, 6], Olson conducted simulation studies on the results of different conditions of test statistics.

In their studies, Todorov and Filzmoser [7] evaluated the performance of Wilks' lambda test statistic in terms of simulated significance levels, power functions, and endurance under various distributions. Gasperik [8] conducted a simulation study to investigate the robustness of the results of MANOVA when the dependent variables had different correlations among different groups and when the sample was taken from the multivariate uniform distribution. With Monte Carlo studies, Adeleke et al. [2] explored the behaviour of three of the existing test statistics (Wilks' lambda, Pillai's trace, and Roy's largest roots) and suggested alternative test statistics to perform MANOVA tests when the normality assumption is violated in the error term. When the MANOVA's assumptions are not achieved for functional data, Górecki and Smaga [9] in their work have proposed permutation tests and random projection tests based on simple function generated from classical test statistics.

In practice, in most cases, it is not possible to sustain all of the assumptions for multivariate analysis of variance. In this case, the question of how Wilks' lambda, Pillai's trace, Hotelling's trace, and Roy's largest root test statistics perform on different conditions and in different distributions to achieve MANOVA tests and lack of a study in the literature involving all of the situations mentioned in this scenario constitute the motivation of this work. Hence, the aim of this study is to investigate how Wilks' lambda, Pillai's trace, Hotelling's trace, and Roy's largest root test statistics are affected in different number of variables and different sample sizes when the normal and homogeneous variance assumptions of the MANOVA method are violated. In other words, it is the examination of whether the tests are reliable (robustness) or not.

## 2. Materials and Methods

In the study, for same groups, with different variable numbers and different sample values, various scenarios for different distributions were constructed where group variances are constant (*σ*_12_ = *σ*_22_ = ⋯ = *σ*_*g*2_) and increasing(*σ*_12_ < *σ*_22_ < ⋯<*σ*_*g*2_). In these scenarios, provided that the number of groups *g* = 3, the number of variables *p* = 2 and *p* = 3, and the number of observations *n* = 10, *n* = 20, and *n* = 50, random numbers are generated from Gamma (4-4-4; 0.5), Gamma (4-9-36; 0.5), Student's *t*(2), and normal (0; 1) distributions. Furthermore, the cases where the number of observations in the groups being balanced and unbalanced are also taken into account. By employing 10000 repetitions in Monte Carlo simulation, Wilks' lambda, Pillai's trace, Hotelling's trace, and Roy's largest root test statistics were calculated, and for each of these tests, type-I error value is calculated. By comparing type-I error values with the nominal value of *α* = 0.05, the hypothesis of “if “*p* < *α*,” equality of the means” is rejected. Simulation study (Mass (Modern Applied Statistics with S′-2017.04.21) and Lestat (a package for LEARNING STATISTICS-20.02.2015) package) was performed using RStudio program language.

### 2.1. Test Statistics

As mentioned earlier, MANOVA examines whether average vectors from two or more groups come from the same sample distribution using appropriate test statistics. A test statistics is used to assess a particular hypothesis through sample data obtained from one or more populations. The hypothesis for the mean vectors is as follows:

(1) $$\begin{array}{c} H_{0}:\mu_{1}=\cdots =\mu_{k}=0,\\ H_{1}:\mu_{i}\neq \mu_{j},\\ i<j,\;i,j=1,2,\ldots ,k. \end{array}$$

The four most common test statistics used in testing this hypothesis are Wilks' lambda [10], Hotelling's trace [11], Pillai's trace [12], and Roy's largest roots [2, 13].

### 2.2. Wilks' Lambda Test Statistic

In the comparison of the mean vectors of *p* number of variables and *g* number of groups, the matrices are expressed as follows:

(2) $$\begin{array}{c} B=\overset{g}{\underset{i=1}{\sum }}n_{i}\left({\bar{x}}_{i}-\bar{x}\right){\left({\bar{x}}_{i}-\bar{x}\right)}^{\prime },\\ W=\overset{g}{\underset{i=1}{\sum }}\left(n_{i}-1\right)S_{i}, \end{array}$$

where *B* represents the total matrix of squares between groups and *W* represents the total matrix of squares within groups, *g* is the number of mean vectors to be compared, ${\bar{x}}_{i}$ is the number of observations for the *i*‐th group, $\bar{x}$ is the general mean vector, *n*_*i*_ is the number of observations for the *i*‐th group, and *S*_*i*_ is the variance-covariance matrix for the *i*‐th group.

The statistic which is defined by Wilks [10] for the first time,

(3) $$\begin{array}{c} \Lambda =\frac{\left|W\right|}{\left|B+W\right|}, \end{array}$$

is the ratio of two matrices to the determinant. The approach to zero of this ratio is indicative of the difference between the mean vectors. Furthermore, for *λ*_*i*_ while *BW*^−1^ is the root of the matrix and *s* is the number of matrices different than zero, Wilks' lambda statistic is given as

(4) $$\begin{array}{c} \Lambda =\overset{s}{\underset{i=1}{\prod }}\frac{1}{1+\lambda_{i}}∼U_{p,g-1,N-g}, \end{array}$$

where *g* is the number of groups, *p* is the number of variables in each group, *N* is the number of observations, *λ*_*i*_ is the *i*‐th root of *BW*^−1^, and *s*=min(*g* − 1, *p*). Test statistic in equation (4) can be denoted as follows [2]:

(5) $$\begin{array}{c} \Lambda =\overset{n}{\underset{i=1}{\prod }}{\left(1+\lambda_{i}\right)}^{-1}. \end{array}$$

The critical value in this method is =((1 − *λ*)(∑*n*_1_ − *g*))/*λ*(*g* − 1). For large samples, the Bartlett approach is preferred instead of this test statistic. As a test statistic for the Bartlett method, *L*=−[(*N* − 1 − (*p*+*g*))/2]ln  Λ equation is used. This shows *χ*^2^ distribution for *p*(*g* − 1) degree of freedom [14]. For multivariate multifactor analysis of variance, significance of Wilks' lambda test statistic can also be done with the help of *F* distribution [15].

### 2.3. Hotelling's Trace Test Statistic

In this statistic which is developed by Hotelling [11] and Lawley [16], *λ*_*i*_'s are calculated from the root of *BW*^−1^ matrix [17]:

(6) $$\begin{array}{c} T=\overset{s}{\underset{i=1}{\sum }}\lambda_{i}. \end{array}$$

If *T* > *χ*_Table[*p*(*g* − 1)];*α*_^2^, then there is a difference between mean vectors. In order to test the *T* statistic, *F* distribution can be used [18].

### 2.4. Pillai's Trace Test Statistic

The test statistic which was introduced by Pillai in 1955 is defined as

(7) $$\begin{array}{c} T=\overset{s}{\underset{i=1}{\sum }}\frac{\lambda_{i}}{1+\lambda_{i}}. \end{array}$$

*F*_*T*_ shows an *F* distribution whose degree of freedom is *s*(2*m* + *s* + 1) and (2*n* + *s* + 1). For *s* = 1, distribution is a full *F* distribution [19]:

(8) $$\begin{array}{c} s=\min\left(g-1,p\right),\\ m=\frac{\left|p-\left(g-1\right)\right|-1}{2},\\ n=\frac{N-p-g-1}{2},\\ F_{T}=\frac{2n+s+1}{2m+s+1}\times \frac{T}{s-T}. \end{array}$$

### 2.5. Roy's Largest Root Test Statistic

If the largest root is denoted by *λ*_max_, Roy's largest root test statistic is introduced by Roy in 1957. This statistic is shown as

(9) $$\begin{array}{c} T=\overset{s}{\underset{i=1}{\sum }}\frac{\lambda_{\max}}{1+\lambda_{\max}}. \end{array}$$

The generated value is compared with the Heck graph with *s*, *m*, and *n* parameters. If the *T* statistic is greater than the Heck graph value, it is said to be that there is a difference between the mean vectors [20].

## 3. Results

**Figure 1:**
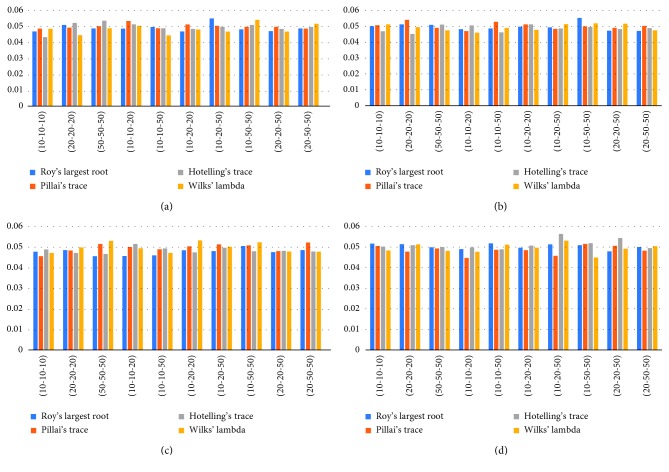
Graphs of type-I error rates of Gamma distribution for constant and increasing variances. (a) Gamma distribution (4,4,4-0.5), *p*=2, homogeneous variance; (b) Gamma distribution (4,4,4-0.5), *p*=3, homogeneous variance; (c) Gamma distribution (4,9,36-0.5), *p*=2, heterogeneous variance; (d) Gamma distribution (4,9,36-0.5), *p*=3, heterogeneous variance.

In Table 1, when we observed type-I error rate of test statistics obtained from the simulation result where the parameter value of the Gamma distribution is (4-4-4; 0.5) and the number of variables is 2 with homogeneous and heterogeneous variances, Pillai's test statistic gives the closest result to the nominal value in balanced and unbalanced sample size. When the number of variables is 3 with homogeneous and heterogeneous variances, Roy's largest root test statistic gives better results in the balanced sample size and Pillai's test statistic gives better results in the unbalanced sample size. In the case of 3 variables, Hotelling's trace test statistic in balanced sample size and Wilks' lambda test statistic in unbalanced sample size give more closer results. In Figure 1, deviations from the type-I error value are expressed visually.

Table 2 shows the type-I error rates of the test statistics obtained from the result of the simulation in case of the degree of freedom of Student's *t* distribution is two. According to the results, in the case of homogeneous and heterogeneous variances with variable numbers 2 and 3, all test statistics give the same results. In the case of homogeneous variance, Pillai's trace test statistic gives the closest result when the sample size is balanced and unbalanced. In the case of heterogeneous variance, Wilks' lambda test statistic gives the closest result to the nominal value when the samples are balanced and unbalanced.

**Figure 2:**
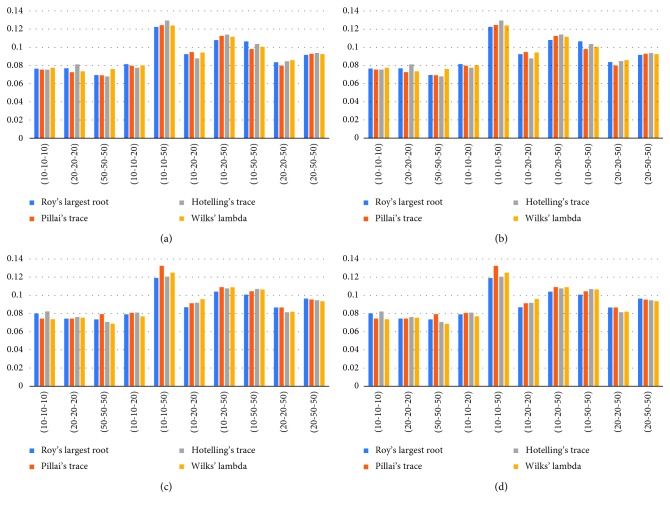
Graphs of type-I error rates of Student's *t* distribution for constant and increasing variances. (a) Student's *t* distribution (2), *p*=2, homogeneous variance; (b) Student's *t* distribution (2), *p*=3, homogeneous variance; (c) Student's *t* distribution (2), *p*=2, heterogeneous variance; (d) Student's *t* distribution (2), *p*=3, heterogeneous variance.

In Figure 2, deviations from the type-I error value are expressed visually. As seen in Figure 2, the largest variation (10-10-50) in the type-I error value is in the group of observation numbers.

In Table 3, in the case of homogeneous variance with the balanced and unbalanced sample size for 2 variables, Roy's largest root test statistic gives the closest results. For 3 variables, Wilks' lambda statistic gives the closest result to the nominal value. In the case of heterogeneous variance, type-I error values show more variability than homogeneous variance situation. Despite this variability, Roy's largest root test statistic gives better results when the number of variables is 2 and 3.

**Figure 3:**
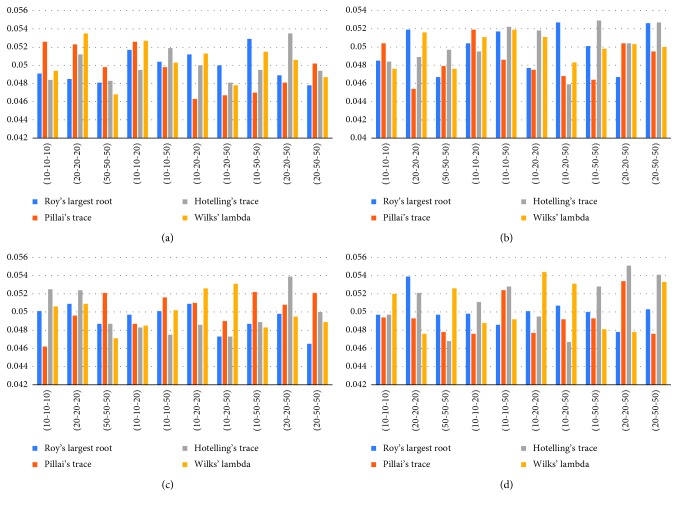
Graphs of type-I error rates of normal distribution for constant and increasing variances. (a) Normal distribution (0,1), *p*=2, homogeneous variance; (b) normal distribution (0,1), *p*=3, homogeneous variance; (c) normal distribution (0,1), *p*=2, heterogeneous variance; (d) normal distribution (0,1), *p*=3, heterogeneous variance.

In Figure 3, deviations from type-I error value are expressed visually.

## 4. Discussion and Conclusion

In this study, the results of the test statistics for different sample sizes were investigated by a simulation study in situations when the MANOVA prerequisites, particularly the normality assumption and homogeneous variance assumption, were violated; upon surveying the literature, the relevant studies in this respect are presented in Section 1. To summarize the results obtained in the previous studies, in his simulation study conducted in 1974, Olsan, who has a lot of studies on this subject, stated that Pillai's trace test statistic gives more robust results than the other test statistics when moved away from the normal distribution and the homogeneity of the covariance matrices is not achieved. In Olson's study in 1976 [5], Roy's largest root test proved contradictory results in almost every case and had a lower power; on the other hand, Olson suggested that Wilks' lambda and Hotelling's trace test statistics could also be used in the case of heterogeneity of the covariance matrices and the degree of freedom of the error matrix was more than 10 times the number of variables. In his study in 1979, Olsen argued that although Pillai's trace test statistic is sometimes less robust than the other test statistics, it is more appropriate to use Pillai's trace test statistic in multivariate hypothesis tests because “the important thing is to keep type-I and type-II errors in balance.” Adeleke et al. [2] proposed two modified test statistic methods (Wilks' lambda, Pillai's trace, and Roy's largest roots) for the case where the assumption of normality is violated. The Monte Carlo results indicated that the modified Pillai's trace and Roy's largest roots test statistics gave relatively robust results, but the original Roy's largest roots statistic gave closer results when the normality assumption was violated in small samples. Moreover, in their study, they suggested that one of the classical methods should be used in case the assumption of normality is satisfied. Górecki and Smaga [9] show that as a result of simulation experiments on the functional data set, the tests do not perform equally well and there is not a single best performing test. Nevertheless, they said that the simple function-based permutation tests obtained from classical test statistics (*W*, *H*, *P*, *R*) are stronger than random projection tests.

The purpose of this study is to conduct a simulation study to compare the performance of various test statistics available for MANOVA on different observations and variable numbers when standard assumptions are violated. According to the results of the study, we can say that, in the case of homogeneous variance, Gamma distribution gives close results to the nominal value (*α* = 0.05) of the test statistics. Especially when the number of variables is 2 with the balanced and unbalanced sample size, it is seen that Pillai's trace test statistic gives more robust results in the homogeneous variance. For 3 variables, Roy's largest root test statistic gives better results than the other test statistics in the balanced sample size. In the case of heterogeneous variance, especially when the variable number is 2, Pillai's trace test statistic gives more robust results. Also, when the variable number is 3, Hotelling's trace test statistic in balanced sample size and Wilks' lambda test statistic in unbalanced sample size give more robust results. In Student's *t* distribution, type-I error of all the test statistics is away from the nominal value. Type-I error rates of all test statistics give the same results between the variable numbers 2 and 3. It can be said that it is not affected by the number of variables. Pillai's trace test statistic in homogeneous variances with balanced and unbalanced sample size and on the other hand Wilks' lambda test statistic in heterogeneous variances with balanced and unbalanced sample size yielded more robust results. In the normal distribution, in the case of homogeneous variance with balanced and unbalanced sample size, Roy's largest root test statistic gives better results for 2 variables and Wilks' lambda test statistic gives better results for 3 variables. Roy's largest root test statistic gives closer results to the nominal value for 2 and 3 variables with balanced and unbalanced samples in heterogeneous variance.

If we generalize the results, for Gamma distribution with homogeneous variance and 2 and 3 variable numbers, Pillai's trace test statistic and with heterogeneous variance Wilks' lambda test statistic give more robust results. In Student's *t* distribution, Pillai's trace test statistic for homogeneous variance and Wilks' lambda test statistic for heterogeneous variance give more robust results. For the normal distribution, for homogeneous variance with variable numbers 2 and 3 Pillai's trace test statistic and for heterogeneous variance Wilks' lambda test statistic give relatively more robust results compared to other test statistics.

In summary, the test statistics used with MANOVA are affected by the violation of the homogeneity and normality assumptions of the covariance matrices, in particular from the unbalanced number of observations. According to scenario results, in the case of homogeneous variance Pillai's trace test statistic and in the case of heterogeneous variance Wilks' lambda test statistic give the best results in terms of performance, or the alternative robust test statistics and Bayes methods, which are recommended in the literature, can be used. This study can be extended by simulation studies for different scenarios with different distributions and parameters.

## Figures and Tables

**Table 1:** Type-1 error rates of simulation results for Gamma distribution.

Number of variables	*p*=2					*p*=3			
Test statistic	*g*	*R*	*P*	*H*	*W*	*R*	*P*	*H*	*W*
*Homogeneous variance*									
Gamma distribution (4-4-4; 0.5)	(10-10-10)	0.0473	0.0491	0.0437	0.0490	0.0501	0.0507	0.0469	0.0513
	(20-20-20)	0.0514	0.0497	0.0527	0.0449	0.0513	0.0541	0.0452	0.0495
	(50-50-50)	0.0492	0.0506	0.0541	0.0493	0.0510	0.0491	0.0511	0.0475
	(10-10-20)	0.0491	0.0539	0.0518	0.0509	0.0482	0.0470	0.0506	0.0460
	(10-10-50)	0.0501	0.0493	0.0493	0.0448	0.0487	0.0529	0.0462	0.0490
	(10-20-20)	0.0473	0.0517	0.0489	0.0485	0.0498	0.0513	0.0513	0.0478
	(10-20-50)	0.0555	0.0508	0.0502	0.0472	0.0494	0.0484	0.0487	0.0514
	(10-50-50)	0.0486	0.0502	0.0514	0.0546	0.0553	0.0500	0.0496	0.0519
	(20-20-50)	0.0475	0.0502	0.0489	0.0472	0.0473	0.0491	0.0483	0.0517
	(20-50-50)	0.0492	0.0491	0.0501	0.0515	0.0471	0.0503	0.0489	0.0475

*Heterogeneous variance*									
Gamma distribution (4-9-36; 0.5)	(10-10-10)	0.0480	0.0458	0.0491	0.0474	0.0517	0.0505	0.0502	0.0483
	(20-20-20)	0.0488	0.0486	0.0474	0.0501	0.0514	0.0478	0.0509	0.0513
	(50-50-50)	0.0458	0.0518	0.0469	0.0533	0.0499	0.0493	0.0500	0.0481
	(10-10-20)	0.0459	0.0503	0.0518	0.0497	0.0490	0.0447	0.0498	0.0477
	(10-10-50)	0.0462	0.0492	0.0496	0.0474	0.0518	0.0487	0.0489	0.0512
	(10-20-20)	0.0487	0.0506	0.0477	0.0535	0.0497	0.0485	0.0507	0.0497
	(10-20-50)	0.0483	0.0516	0.0499	0.0504	0.0513	0.0457	0.0564	0.0531
	(10-50-50)	0.0508	0.0514	0.0485	0.0528	0.0509	0.0515	0.0519	0.0449
	(20-20-50)	0.0477	0.0483	0.0484	0.0481	0.0479	0.0506	0.0544	0.0492
	(20-50-50)	0.0488	0.0525	0.0481	0.0480	0.0500	0.0482	0.0495	0.0504

**Table 2:** Type-1 error rates of simulation results for Student's *t* distribution.

Number of variables	*p*=2					*p*=3			
Test statistic	*g*	*R*	*P*	*H*	*W*	*R*	*P*	*H*	*W*
Homogeneous variance	(10-10-10)	0.0765	0.0754	0.0754	0.0774	0.0765	0.0754	0.0754	0.0774
	(20-20-20)	0.0769	0.0726	0.0812	0.0735	0.0769	0.0726	0.0812	0.0735
	(50-50-50)	0.0695	0.0692	0.0680	0.0760	0.0695	0.0692	0.0680	0.0760
	(10-10-20)	0.0815	0.0796	0.0775	0.0802	0.0815	0.0796	0.0775	0.0802
	(10-10-50)	0.1224	0.1244	0.1294	0.1240	0.1224	0.1244	0.1294	0.1240
	(10-20-20)	0.0924	0.0947	0.0878	0.0942	0.0924	0.0947	0.0878	0.0942
	(10-20-50)	0.1079	0.1125	0.1140	0.1116	0.1079	0.1125	0.1140	0.1116
	(10-50-50)	0.1065	0.0980	0.1036	0.1005	0.1065	0.0980	0.1036	0.1005
	(20-20-50)	0.0836	0.0799	0.0847	0.0859	0.0836	0.0799	0.0847	0.0859
	(20-50-50)	0.0916	0.0928	0.0937	0.0924	0.0916	0.0928	0.0937	0.0924

Heterogeneous variance	(10-10-10)	0.0802	0.0744	0.0823	0.0737	0.0802	0.0744	0.0823	0.0737
	(20-20-20)	0.0744	0.0744	0.0761	0.0754	0.0744	0.0744	0.0761	0.0754
	(50-50-50)	0.0735	0.0793	0.0707	0.0687	0.0735	0.0793	0.0707	0.0687
	(10-10-20)	0.0791	0.0807	0.0810	0.0768	0.0791	0.0807	0.0810	0.0768
	(10-10-50)	0.1191	0.1324	0.1205	0.1249	0.1191	0.1324	0.1205	0.1249
	(10-20-20)	0.0869	0.0913	0.0918	0.0958	0.0869	0.0913	0.0918	0.0958
	(10-20-50)	0.1041	0.1090	0.1076	0.1089	0.1041	0.1090	0.1076	0.1089
	(10-50-50)	0.1007	0.1044	0.1069	0.1064	0.1007	0.1044	0.1069	0.1064
	(20-20-50)	0.0866	0.0866	0.0814	0.0820	0.0866	0.0866	0.0814	0.0820
	(20-50-50)	0.0964	0.0954	0.0946	0.0935	0.0964	0.0954	0.0946	0.0935

**Table 3:** Type-1 error rates of simulation results for normal distribution.

Number of variables	*p*=2					*p*=3			
Test statistic	*g*	*R*	*P*	*H*	*W*	*R*	*P*	*H*	*W*
Homogeneous variance	(10-10-10)	0.0491	0.0526	0.0484	0.0494	0.0485	0.0504	0.0484	0.0476
	(20-20-20)	0.0485	0.0523	0.0512	0.0535	0.0519	0.0454	0.0489	0.0516
	(50-50-50)	0.0481	0.0498	0.0483	0.0468	0.0467	0.0479	0.0497	0.0476
	(10-10-20)	0.0517	0.0526	0.0495	0.0527	0.0504	0.0519	0.0495	0.0511
	(10-10-50)	0.0504	0.0498	0.0519	0.0503	0.0517	0.0486	0.0522	0.0519
	(10-20-20)	0.0512	0.0463	0.0500	0.0513	0.0477	0.0475	0.0518	0.0511
	(10-20-50)	0.0500	0.0467	0.0481	0.0478	0.0527	0.0468	0.0459	0.0483
	(10-50-50)	0.0529	0.0470	0.0495	0.0515	0.0501	0.0464	0.0529	0.0498
	(20-20-50)	0.0489	0.0481	0.0535	0.0506	0.0467	0.0504	0.0504	0.0503
	(20-50-50)	0.0478	0.0502	0.0494	0.0487	0.0526	0.0495	0.0527	0.0500

Heterogeneous variance	(10-10-10)	0.0501	0.0462	0.0525	0.0506	0.0497	0.0494	0.0497	0.0520
	(20-20-20)	0.0509	0.0496	0.0524	0.0509	0.0539	0.0493	0.0521	0.0476
	(50-50-50)	0.0487	0.0521	0.0487	0.0471	0.0497	0.0478	0.0468	0.0526
	(10-10-20)	0.0497	0.0487	0.0483	0.0485	0.0498	0.0476	0.0511	0.0488
	(10-10-50)	0.0501	0.0516	0.0475	0.0502	0.0486	0.0524	0.0528	0.0492
	(10-20-20)	0.0509	0.0510	0.0486	0.0526	0.0501	0.0477	0.0495	0.0544
	(10-20-50)	0.0473	0.0490	0.0473	0.0531	0.0507	0.0492	0.0467	0.0531
	(10-50-50)	0.0487	0.0522	0.0489	0.0483	0.0500	0.0493	0.0528	0.0481
	(20-20-50)	0.0498	0.0508	0.0539	0.0495	0.0478	0.0534	0.0551	0.0478
	(20-50-50)	0.0465	0.0521	0.0500	0.0489	0.0503	0.0476	0.0541	0.0533

## Data Availability

The [simulated data] data used to support the findings of this study are available from the corresponding author upon request.
